# The effects of MCH insurance cards on improving equity in access and use of maternal and child health care services in Tanzania: a mixed methods analysis

**DOI:** 10.1186/s41043-016-0075-8

**Published:** 2016-11-18

**Authors:** August Kuwawenaruwa, Gemini Mtei, Jitihada Baraka, Kassimu Tani

**Affiliations:** Ifakara Health Institute, Plot 463, Kiko Avenue Mikocheni, P.O. Box 78 373, Dar es Salaam, Tanzania

**Keywords:** Insurance, Demand-side financing, Equity, Place of delivery, Targeting mechanism, Tanzania

## Abstract

**Background:**

Inequity in access and use of child and maternal health services is impeding progress towards reduction of maternal mortality in low-income countries. To address low usage of maternal and newborn health care services as well as financial protection of families, some countries have adopted demand-side financing. In 2010, Tanzania introduced free health insurance cards to pregnant women and their families to influence access, use, and provision of health services. However, little is known about whether the use of the maternal and child health cards improved equity in access and use of maternal and child health care services.

**Methods:**

A mixed methods approach was used in Rungwe district where maternal and child health insurance cards had been implemented. To assess equity, three categories of beneficiaries’ education levels were used and were compared to that of women of reproductive age in the region from previous surveys. To explore factors influencing women’s decisions on delivery site and use of the maternal and child health insurance card and attitudes towards the birth experience itself, a qualitative assessment was conducted at representative facilities at the district, ward, facility, and community level. A total of 31 in-depth interviews were conducted on women who delivered during the previous year and other key informants.

**Results:**

Women with low educational attainment were under-represented amongst those who reported having received the maternal and child health insurance card and used it for facility delivery. Qualitative findings revealed that problems during the current pregnancy served as both a motivator and a barrier for choosing a facility-based delivery. Decision about delivery site was also influenced by having experienced or witnessed problems during previous birth delivery and by other individual, financial, and health system factors, including fines levied on women who delivered at home.

**Conclusions:**

To improve equity in access to facility-based delivery care using strategies such as maternal and child health insurance cards is necessary to ensure beneficiaries and other stakeholders are well informed of the programme, as giving women insurance cards only does not guarantee facility-based delivery.

## Background

High maternal mortality is a major public health concern. The maternal mortality ratio in developing countries is 230 per 100,000 live births versus 16 per 100,000 live births in developed countries [1], and more than half of these deaths occur in sub-Saharan Africa [2]. Tanzania has a high rate of maternal death; in 2010, the maternal mortality ratio was 454 per 100,000 live births [3].

Many maternal deaths are preventable. Most are caused by factors attributed to delays in seeking care, lack of skilled birth attendants, and poor quality of health services [4–7]. More than 80% of deaths can be prevented if pregnant women access essential maternity care and are attended by a skilled provider at childbirth [2, 8, 9].

Inequity in access to and use of child and maternal health services is impeding progress towards the maternal and child health Millennium Development Goals [2, 10]. Statistics from the 2010 Tanzania Demographic and Household Survey (TDHS) show that women in the highest wealth quintile were far more likely to deliver in a facility (90%) than women in the lowest quintile (33%), who were also less likely to have received four or more antenatal care visits and to receive timely postnatal care [11].

To address either low usage or disparities in usage of maternal and newborn health services, many countries have adopted demand-side financing interventions to influence access, use, and provision of reproductive health services [12]. Such demand-side financing interventions include voucher schemes [13, 14], social health insurance, and conditional cash transfers [15, 16]. Social health insurance schemes in particular provide strong incentives on the demand side since they often inform, guide, and empower the clients to seek care from the accredited health facilities. Demand-side financing is geared towards reducing financial barriers faced by women when seeking health care services from formal health care providers [17, 18].

**Fig. 1:**
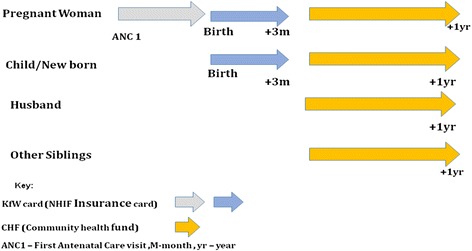
Programme design—Mbeya/Tanga Region. The figure shows how the programme was designed; women use the insurance card during pregnancy and up to 3 months after delivery. Thereafter, the husband and four dependants of the pregnant woman are issued with a free 1-year Community Health Fund (CHF) membership card, paid for by the programme funds

In 2010, Tanzania’s National Health Insurance Fund (NHIF) introduced a pilot programme of maternal and child health insurance cards (MCH card) in Rungwe district in the Mbeya region of Tanzania. Two districts were selected for the pilot programme, and the intervention was subsequently scaled to the entire region. The programme aimed to provide free insurance cards to poor women and their families in order to enhance financial protection. The insurance card can be used during pregnancy and up to 3 months after delivery. The husband and four dependants of the pregnant woman were also issued with a free 1-year Community Health Fund (CHF) membership card, paid for by the programme funds (Fig. 1). The MCH insurance cards transfer purchasing power to the poor who choose which facility they will visit, and providers are reimbursed for their services from the NHIF. Cardholders receive a package of essential maternal health services, as well as treatment of pregnancy- and delivery-related complications.

Initially, Rungwe was included in an individual targeting study arm in which eligibility was determined based on each woman’s socioeconomic status. However, after approximately 2 years, individual targeting in the area was replaced with geographic targeting, in which all women living in a geographic area were eligible for the MCH card.

This paper presents a case study of the Rungwe district, which aimed to examine in-depth the experiences of women and health care providers with a maternal and child health insurance scheme, focusing on delivery location. Specifically, it examines the extent to which the use of the MCH cards was equitable, and it also identifies factors that influenced women’s decisions regarding use of the MCH card and the choice of delivery location.

## Methods

### Study design

This case study used a mixed methods approach. We conducted a quantitative assessment of facility use in a sample of the population in Rungwe district and whether the most disadvantaged women, as indicated by their educational status, had taken advantage of the MCH cards to deliver in a facility. Use of a qualitative method enabled the research team to explore women’s decisions on birth place, birth experience itself, and use of the MCH card.

### Study area

The Rungwe district in Mbeya region was purposively selected for data collection. It was the only district in the region that had implemented individual targeting before adopting geographic targeting, and thus, it offered not only the opportunity to describe women’s and providers’ experiences with the current geographic targeting strategy but also explores providers’ attitudes towards both strategies and the reasons for abandoning individual targeting. Data related to the switch from individual targeting to geographical targeting will be reported in a second paper [19].

### Sampling technique

Purposive sampling was used to select representative facilities at the district, ward, facility, and community level. The district hospital and both health centres were included, and four dispensaries were selected based on physical accessibility, enrolment rate to the MCH insurance card, and being served by a health centre. Thus, a total of seven health care facilities and their catchment areas were included in the study.

### Quantitative data collection

No routine data were available from medical or programme records to evaluate the extent to which more disadvantaged women were delivering in facilities compared to their less disadvantaged peers. The health care facilities were visited, and the researchers reviewed the MCH card beneficiary register book for the women who had delivered between January and December 2013. All the beneficiaries were listed on a paper as they appear in the register book at the facility. Thereafter, a systematic random sampling was used, whereby the team leader identified a starting point and subsequent beneficiaries were identified at an interval of five. A total of 30 women from each dispensary, 20 women from each health centre, and 40 women from the hospital were identified for an interview. Their telephone numbers were obtained from the registry, and the research team had to set appointments for a telephone survey with the beneficiaries on the same day or a day after.

### Qualitative data collection

To assess the factors influencing women’s use of the MCH card and decision-making regarding delivery location, two women, one who had the MCH card and delivered at home and one who had the card and delivered at a facility, were randomly selected from the register at each facility. Interviews were conducted at the women’s homes. The research team also conducted interviews during the facility visit with purposively selected health workers, traditional birth attendants, and village leaders.

A total of 31 in-depth interviews (IDIs) were conducted in September 2014. Interview guides contained a range of topics in relation to the decision about place of birth, knowledge about the insurance scheme, and recommendations about maternal and child health services. Interview guides were created in English and subsequently translated into Kiswahili by the bilingual research team and research assistants, who also conducted the interviews. The research team conducted interviews in pairs: one facilitated the interview while the other took notes. All interviews were also digitally recorded, and the audio files were transcribed and translated by a research assistant. In addition, the researchers cross-checked the audio files and transcripts for data quality assurance.

### Data analysis

#### Equity analysis

To assess equity, three categories of education levels were used: no education (did not attend school or dropped out before completing basic primary school), primary school (basic primary school education up to standard seven), and secondary school (secondary education and above). A comparison of the beneficiaries’ education was compared with the educational distribution of women who had delivered a child in the previous 5 years from the Tanzania Demographic Household Survey (TDHS) of 2010 and a household survey conducted in one of the districts in Mbeya by Ifakara Health Institute in February 2014. Microsoft Excel was used for the analysis.

#### Qualitative analysis

A thematic analysis approach was adopted. Two research scientists read each transcript independently and developed a final code book. A brief discussion was held by the researchers to agree on the final themes. The team worked together and coded a few transcripts together to ensure consistency and then each worked independently on the remaining transcripts. The team then combined and discussed the coded transcripts and identified themes and appropriate quotations for the manuscript. Data were analysed using NVivo 10 software.

#### Ethical approval and consent

Ethical clearance for this study was obtained from the Ifakara Health Institute Institutional Review Board (IRB) IHI/IRB/No 12-2014. Written consent was sought from the study participants after the team leader had explained the objectives of the study. Participants were informed that their participation was voluntary and at any time, they had the right to withdraw without any penalty. The field team assured participants about confidentiality of all information throughout the study. Interviews were conducted in the local language (Kiswahili) and tape recorded with the permission of the study participants.

## Results and discussion

### Quantitative results

**Fig. 2:**
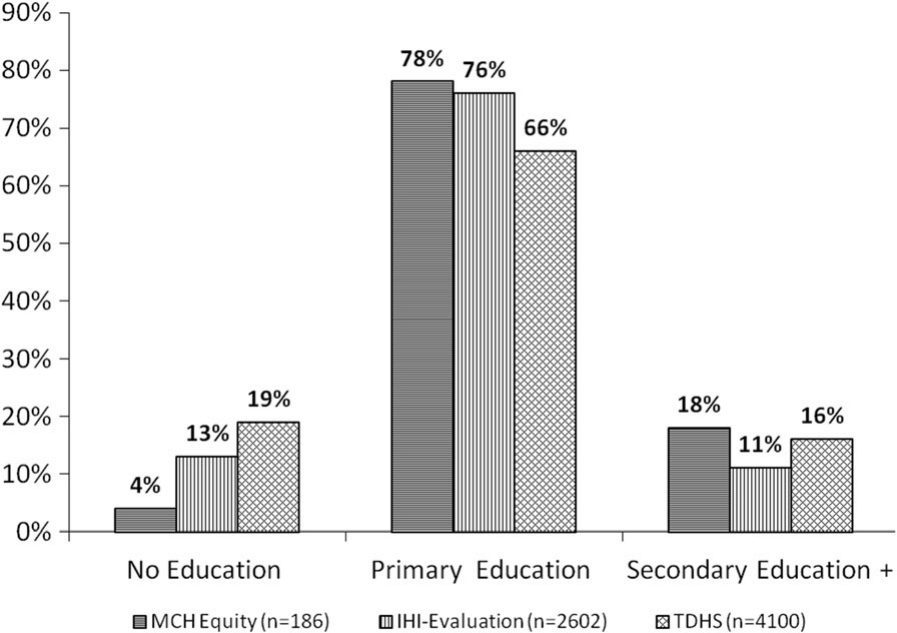
Educational distribution for women of reproductive age reported to deliver at health facilities. Presents the findings on equity based on educational attainment and reported facility delivery. The findings were compared with the educational distribution of women from the Mbarali district from the 2010 Tanzania Demographic and Household Survey (TDHS) and from the Ifakara Health Institute (IHI) evaluation of the MCH health insurance card programme (Bima Wazazi) in Mbarali conducted in 2014

Of the 200 women in the sample, 190 (95%) were interviewed, including 37 from the district hospital registry, 38 from the health centres, and 115 from the dispensaries. Figure 2 presents the findings on equity based on educational attainment and reported facility delivery. Compared with the educational distribution of women from the Mbarali district from the 2010 TDHS and from the IHI evaluation of the MCH health insurance card programme (Bima Wazazi) in Mbarali conducted in 2014, women with low educational attainment were under-represented amongst those who reported having received the MCH card and used it for delivery at a facility. Women with no education accounted for 19 and 13% of the women in the TDHS and IHI populations, respectively, compared with 4% in our sample of women who had received the MCH card.

### Qualitative results

#### Decision about place of birth

Decision about where to deliver is influenced by several factors, which have been categorized as individual factors, financial factors, and health system factors.

#### Individual factors

A number of factors influenced individual decision-making on where to deliver; these included complications during previous pregnancy, problems during the current pregnancy, delay in decision-making, and male involvement in decision-making.

### Complications during previous pregnancies/deliveries

Having experienced or witnessed problems during previous pregnancies influenced women’s decision-making about birth location. Some women pointed out that they had to go to the facility because during their first delivery they experienced delivery complications or worried about the risk of complications, leading them to choose the health care facility for safe delivery. Additionally, women who had previously experienced problems were in some cases advised to stay closer to the facility to be able to access care quickly when labour began.

…..for example I saw some women delivering at home and they faced a lot of problems… You will be surprised, some of them died and for the other women their babies died, but delivering at the hospital is better because I have delivered all my the three children at the hospital and I did not face any problem…. (IDI, with women delivered at facility, 2014)

Similarly, women’s experience in delivering at the health facility influenced their decision-making for subsequent pregnancies. Interviews revealed that experience in birth varied for women who had given birth once compared to those who had given birth more than once. Women who had given birth both at home and at the facility were in a position to make comparison on both services. The benefits to facility-based deliveries mentioned included opportunities to get health education, increased recognition of danger signs, and increased confidence of women to discuss birth issues with health care providers. Some women reported feeling safer delivering at a health facility.

The difference I saw when I delivered the first child at the health facility is that they helped me very much because the baby had problem with eyes focus and they advised me that the child’s vision is not good and they told me to take the child to xxx hospital as there were no medicines, so the health provider noticed the problem before I was discharged while at home it could have been difficult to notice. (IDI, with women delivered at home, 2014)

Another woman pointed out that

…the experience which I had after delivering this child at the facility is that delivering at the facility is more safe/secure because I have seen people who are delivering at home they face a lot of problems/difficulties…. (IDI, with women delivered at facility, 2014)

### Complications during current pregnancy

Complications during pregnancy also influenced women’s choice on where to deliver. Some women who reported health problems during pregnancy (e.g. suffering from pain, fever, and swollen legs) delivered at home because their condition prevented them from walking to the facility.

Regardless of delivery location, the majority of women expressed concerns about complications during delivery that could not be addressed at home. Respondents highlighted that delivering at the health care facilities is safer because at the health care facility the staff could identify danger signs and assist them.

### Delay in decision-making

Amongst women who did not deliver in health facilities, delay in going to the facility was cited as a reason for delivering at home, as evidenced by the following respondent:

I delivered at home because I was late to go to the facility, if I had left at home the time I felt the pain I would have arrived earlier at the facility…But as my waist was paining I failed to walk that is why I called a cyclist so that he could take me, but he found I had already delivered the baby when he arrived.. (IDI, with women delivered at home, 2014)

### Male involvement in decision-making on place of delivery

Study participants were asked whether their partners and other relatives influenced the woman’s decision to deliver at the health care facility or at home. In some cases, there was lack of communication and family support, while some women who had delivered at the facilities received support from their partners, which took the form of encouraging facility delivery and accompanying the woman to a facility. One respondent pointed out that

…..As I was suffering from fever… I told him that I am supposed to go and deliver at the facility and he said I cannot let you deliver at home, we must go to the health care facility… (IDI, with women delivered at facility, 2014)

Another one said that

..As I felt the labour pains, I told my husband to call my mother so that we may go with her to hospital, he called my mother and she came and we went with her to the facility… (IDI, with women delivered at facility, 2014)

… I as I felt the labour pains, he said lets go to hospital… (IDI, with women delivered at facility, 2014)

However, some women reported delivering at home because their husbands were not at home when labour pains started. One respondent highlighted saying that

…when labour pains started my husband was at farm, I sent the child to tell the neighbours to call him, and take the car which I will use to go to hospital, by the time they arrived they found I have already delivered… (IDI, with women delivered at home, 2014)

### Financial factors

Findings show that a number of financial factors influenced a woman’s choice about where to give birth.

#### Cost of transportation

Participants talked about distances to the health facilities that resulted in incurring costs for transport. Women who delivered in facilities reported using motorcycles as a means of transport to the health care facility. Many women who delivered at home cited that transport cost was what led them to deliver at home as they could not afford transport.

….Indeed it’s far to go to the dispensary since our area is situated with hills but we use motorcycle to get there, we pay about 10,000 shillings (approx. 4-5USD) but it depends on the distance…. (IDI, with women delivered at home, 2014)

#### Other costs associated with facility deliveries

Facility cost was mentioned as one of the factors for home delivery. In most cases, facilities are distant from villages and there are costs associated with being far from home, such as costs for food for the pregnant women and care giver(s) and accommodation. Therefore, for some women, it was difficult to accumulate adequate capital, and as a result, they opted to deliver at home. One respondent, speaking about how the referral she received influenced her decision about where to deliver, stated the following:

It might influence, because you have told me to go and deliver at xxxx facility, I don’t have the bus fare to xxx, and where will I stay in there? After all I don’t have relatives there and for the time I will be in xxxx facility what I am I going to eat? (IDI, with women delivered at home, 2014)

#### Seasonal variability in income

Financial access appeared to vary over time. Some respondents discussed the difficult situations in their communities created by their dependence on cocoa; in the off-season, families that had experienced poor harvests faced difficulties in accessing the health facilities as they have no money to hire transport. Those who had been referred to the hospital level for delivery faced additional financial hardships, as mentioned above.

#### Imposition of fines for home delivery

Some health facilities reported that they levied fines on women who deliver at home. The imposition of the fines at the health facilities, which ranged from TZS 5000 to TZS 10,000 (approx. 2–5 USD) from one village to the other, was meant to encourage more women to visit the health facility for delivery. Interviews with women and village executives revealed that the money is being paid to the health facility when the woman comes for postnatal services, and the money collected was placed directly in the health facility account to help other facility operations.

Some participants reported that the imposition of the fees has helped to reduce home deliveries as the women are afraid of being fined. They also reported that if a woman delivered at home, she has to pay the fine when she comes to the health facility for weight monitoring and immunization for the newborn.

…….the majority do deliver here at the dispensary but they have realized that if they do not deliver at the health care facility they have to pay five thousand as a fine and at the beginning it was two thousand and right now it’s five thousand, the majority have been sensitized and they deliver at the dispensary…….. (GD, Health facility provider, 2014)

Information on imposition of fines was known to the other community members. One respondent was informed by her mother-in-law to go to the facility as they will be charged in case she delivers at home:

…we must go at the hospital because now days if you do not deliver at the health facility you will be fined, ……..I accepted to go to the facility, but as we were waiting for the motorcycle I delivered.. (IDI, with women delivered at home, 2014)

#### Health insurance (MCH insurance card)

The MCH insurance card was meant to address financial barriers in access and use of maternal and child health care services in the study area. During the interviews, women were asked whether having the MCH insurance card influenced their decision to go to the facility. Women had learned about insurance during ANC visits, and some were aware that with the MCH insurance card they would receive free health care whenever they fall sick. A few women acknowledged that the insurance card had motivated them to deliver at the health care facility and explained that without the health card they could not afford the cost of facility delivery.

…..yes, it has motivated me a lot because I know I will get good services just for free with the card… (IDI, with women delivered at facility, 2014)

Women also reported that in the past they were advised to bring birth preparedness materials such as sterile gloves when coming for delivery, but after the implementation of the MCH insurance cards, they were no longer told to bring anything other than clean clothes for the baby.

Major changes in seeking care were observed at the hospital level as implementers noted that the MCH insurance card had induced women to seek maternal and child health care services at the higher level facilities. Regional level implementers experienced an increase in claim forms, which shows that more women are seeking health care. Based on the number of claim forms received and facility visits, they have realized that more women are seeking health care at the hospital level compared to the lower level facilities.

…women are now sleeping on the floor due to shortage of beds because they know the hospital is good and the services are free, so everyone is going to the hospital to seek health care services… (GD, with regional implementer, 2014)

### Health system factors

#### ANC visits

Findings show that most of the respondents visited a health care facility for ANC services; they started ANC care in their second to fifth month of pregnancy and attended three to six ANC visits before delivery.

Some women reported that individual educational awareness on danger signs received during ANC visits influenced their decisions to go to a health care facility for delivery. One respondent highlighted that

…First of all we are sensitized that pregnant women are supposed to deliver at the health care facility so we have seen it’s better to come here and we appreciate for the health education given as we have seen very few women or new-borns die… (IDI, with women delivered at facility, 2014)

Health facility staff who were interviewed confirmed that they usually educate and encourage women to come and deliver at the health care facility. They reported that they provide education on nutrition, what women can do and what they should not to do while at home, danger signs associated with pregnancy, and birth preparedness.

#### Providers and client relationship

The majority of interviewed women felt that they were well treated at the facilities, though a few women pointed out that they were mistreated by the staff and that discouraged them from delivering at a facility.

..I was not pleased with the staff the one who was attending me because [she] was so harsh, was as if [she] has never given birth, she is my fellow woman and does know the labour pains but I was not pleased. Till today even if I go to the hospital as I see her I feel very bad….. (IDI, with women delivered at facility, 2014)

Another participant highlighted saying

…but I thank God when I gave birth, the nurses who attended me were very good …. Treated me nicely ….they motivated me that next time I should give birth at the facility ….. Language was good and I liked the environment…. (IDI, with women delivered at facility, 2014)

#### Traditional birth attendants

During the field work, we were interested to know the role of the traditional birth attendants in the community and decision to choose the delivery place for women. Out of all women interviewed, only few were assisted by traditional birth attendants (TBAs) during birth, as the TBAs are discouraged from providing delivery services at home and instead are encouraged to advise the woman to deliver at the health facility. One of the reasons given for not using the TBA was unhygienic practices; some women noted that the same tools and equipment were used for different women which could lead to infection. One woman highlighted

..In the past we were told go and deliver at the traditional midwife but right now we have stopped, people are not delivering to the traditional midwives. You will be surprised that same mat is used for several deliveries and just the same razor. People realized that they will be infected with various diseases through that way, so now days have stopped… (IDI, with women delivered at facility, 2014)

Interviews with TBAs showed that they had been assisting women to give birth in the past but are no longer assisting them, except in the very rare case when it happens that women fail to go to the facility for delivery.

……we have been advised by the facility in charge to bring the women at the health facility, sometimes families do come at my house to ask for assistance to go and assist delivery at their homes and when you reach at the woman’s house, you find the baby’s head is already out, so what you do is to assist to take the baby out and advise them to take the baby to the health facility and most of the times I do escort them …. (IDI, with TBA, 2014)

### Discussion

The case study used a mixed methods approach to examine the extent to which women with low educational attainment were represented amongst women delivering in facilities in the study area and to examine factors influencing women to use or not to use the MCH card for delivery and women’s decisions about the location of delivery.

In terms of equity in access to facility-based delivery care, as assessed by educational level, women with no education are under-represented in the sampled population of women who delivered in health facilities in the study region. This could have been an issue of access, including lack of funds to pay for transport and for the ancillary costs of food and local lodging, but could also be attributable to the comparison group used in the analysis. Although the TDHS and IHI evaluations demonstrated higher percentages in the no education category, the population living in the study district may have higher educational attainment than the TDHS and IHI evaluations, which were done on larger populations and, in the case of TDHS, 4 years earlier.

In terms of women’s attitudes and behaviours, our study revealed that many women interviewed had planned to give birth at a health facility even before the distribution of the MCH cards. Their plans were based on the education rendered during antenatal care visits to the health care facilities, although a local policy in which fines were levied on women delivering at home may have also influenced their choice. Majority of the women interviewed highlighted the advantages of facility-based delivery, particularly related to antenatal and intra-partum complications. Decision on the place of birth was influenced by the availability of means and cost of transport, as well as health system and financial factors, as has been reported elsewhere [11, 20, 21].

In our study, women played an important role in the decision-making process about where to deliver, although husbands also had an important role in deciding the place for delivery. This contrasts a previous study conducted in a coastal region, in which it was reported that husbands were the primary decision-makers. Other family members also played a role in decision-making if needed or if there was disagreement about a husband’s decision [22]. A recent qualitative study conducted in Ethiopia has shown that decision-making about the place of delivery was initiated by the woman or the husband [23]. Another study, conducted in Sierra Leone, on decision-making about delivery location revealed that individuals rarely made decisions alone about where to deliver and with whom assisting [24, 25]. Instead, these decisions were made in conjunction with older women, especially the TBAs or female family members. Male family members were mostly responsible for providing money and transport rather than being involved directly in discussions [22, 24]. Similar to our findings, a study by Treacy indicated that problems during previous deliveries and perceptions and expressions of bodily symptoms, as well as the interpretation of different risks, influenced decision-making about place of birth [21, 24].

In our study, the role of the TBAs differed from other settings in which women relied heavily on TBAs to assist them during delivery [11]. In Rungwe, TBAs are discouraged from assisting women to deliver at home and are encouraged to bring women to facilities for delivery. Giving TBAs a small allowance and penalizing women who had home delivery was used as a mechanism to reduce home delivery cases. A similar transition in the role of TBAs occurred in certain municipalities in the Department of Huehuetenango in Guatemala where a community-centred birthing house model was established [26]. In this area, TBAs were encouraged to accompany women to the birthing centres, which are staffed by trained nurses, and to participate in the birthing process. TBAs in this setting were supportive of the model and played an instrumental role in acceptance and use of the birthing homes by the population [26].

Our study demonstrated that despite the challenges in the implementation of the health insurance card scheme and the low awareness of beneficiaries about its benefits, women were pleased that they are no longer incurring costs at the health care facilities. Factors that still appear to impede women from seeking facility delivery include transport, which still incurs costs and may not be readily available, and the poor treatment they have received at facilities in the past [27].

The availability of motorcycle transport, as mentioned by many of the women interviewed, has reduced the shortage of transportation experienced in the past. Despite this, transportation remains an issue in our setting that impedes access to facility deliveries, as in many other settings [21, 22, 25]. A study in the western part of Tanzania found that providing transport to either a health centre or a hospital had minimal impact on the percentage of women preferring to deliver at home [28]. This could suggest that removing the barriers of availability and cost of transportation is not necessarily sufficient to overcome other factors that influence women to deliver at home.

Several factors related to the quality of care at facilities influenced women’s decision-making. One dimension of this was a lack of respectful care at facilities. We found that psychological abuse experienced during previous facility births had an impact in deciding whether to use or not to use health care facility for delivery. Physical and psychological abuse has similarly been reported as a deterrent to choosing a facility-based delivery in the Maasai population in southern Kenya [29]. In Ethiopia, women who had similar experiences preferred to deliver at home [21, 24, 30]. Another dimension of quality of care that influenced women’s decision-making was health system bottlenecks, such as the shortage of drugs and other medical supplies in the facilities [23, 28]. Addressing quality of care issues may incentivize a further increase in the rate of facility delivery.

### Limitation of the study

Our study has several limitations. First, the quantitative data to assess the extent to which the programme reached those most in need was based on women attending the purposively sampled hospital, health centres, and dispensaries, which may not be representative of all women living in the district. The data were collected by telephone, and although most women had access to a phone and about 95% were reached, those who were contacted may differ from those who were not contacted. This would likely bias our results to underestimating the number of women with low education who delivered in a facility. Finally, the data collected was limited to the woman’s education, which is not a highly exact indicator of her family’s economic status. In the study area, coverage of facility delivery for the sampled seven facilities was so high (98.0%), it was difficult to identify women, particularly those with low educational attainment, who had not delivered in a facility to assess their experiences.

## Conclusions

In order to improve equity in access to facility-based delivery care using strategies such as the MCH insurance card, it is necessary to ensure beneficiaries and other stakeholders are well informed of the programme. Consideration of other factors which might facilitate or hinder the achievement of the programme goals should also be examined, as giving pregnant women MCH insurance cards alone is not sufficient to guarantee facility-based delivery. Future research might look at programmes which intend to address multiple barriers together rather than a single barrier.

## Abbreviations

ANC: Antenatal care
CHF: Community Health Fund
GD: Group discussion
KfW: German Development Bank
NHIF: National Health Insurance Fund
TBA: Traditional Birth Attendant
